# Theoretical Calculation Study of Nanomaterials

**DOI:** 10.3390/nano16160970

**Published:** 2026-08-07

**Authors:** Yuhua Duan, J. Woods Halley

**Affiliations:** 1 National Energy Technology Laboratory, United States Department of Energy, Pittsburgh, PA 15236, USA; 2 School of Physics and Astronomy, University of Minnesota, Minneapolis, MN 55455, USA; halle001@umn.edu

## Introduction

With the innovation of high-performance computers, it has become possible in this day and age for first-principles density functional theory (DFT) calculations to handle large atomic systems with hundreds of atoms, paving ways to explore properties of nanomaterials for many applications. Generally, nanomaterials are defined as materials possessing, at minimum, one external dimension measuring 1–100 nm, which means that at least half of the particles in a given sample must measure 100 nm or below. Within this size range, nanoparticles may contain anywhere from fewer than one hundred to several thousand atoms. However, without further approximation (e.g., tight binding), current first-principles approaches still cannot calculate the properties of nanoparticles approaching the ~100 nm scale, in part due to computational complexity (N^3^ scaling) and the limits of Moore’s law. Despite these computational challenges, recent advancements have emerged. The new methods including first principles-based ab initio molecular dynamics, machine learning (ML), and artificial intelligence (AI)-related techniques have been employed widely to bridge the gap between accessible DFT scales and nanoscale.

In addition to theoretical developments, practical applications of these calculational techniques to understand and design nanomaterials are already widespread across diverse industries, including healthcare, cosmetics, environmental preservation, and air purification. For instance, in healthcare, nanomaterials are used for targeted drug delivery, and first-principles calculations help predict their interactions with biological molecules. In environmental preservation, these calculations assist in designing nanomaterials that can efficiently capture pollutants or catalyze chemical reactions. As computational methods continue to improve, first-principles calculations will further enable the discovery of new applications for nanomaterials.

Between 2022 and 2026, we edited two Special Issues for *Nanomaterials* [1,2]. The first issue [1] collected 19 articles highlighting the first-principles calculation study of nanomaterials [3,4,5,6,7,8,9,10,11,12,13,14,15,16,17,18,19,20,21]. The 2nd edition on theoretical calculation of nanomaterials [2] contains17 articles [22,23,24,25,26,27,28,29,30,31,32,33,34,35,36,37,38]. These publications cover a wide range of applications through theoretical simulations. The methods employed span atomistic calculations to ML as well as finite element approaches. Here, we organize these publications into six themes. Table 1 then summarizes major topics and methods as well as software used in these studies. We conclude with some suggestions for possible directions for future work.

**Theme 1: Spintronics and Correlated Electronic Structure**

The following papers explored spin–orbit coupling, phase transitions, and low-dimensional confinement to engineer quantum-state electronics.

- Samanta et al. [6] employed first-principles dynamical mean-field theory (DMFT) to investigate the correlated electronic structures of the layered honeycomb magnets α-RuCl_3_ and α-RuI_3_. The results suggest a potential realization of correlated flat bands with strong spin–orbit coupling and a quantum spin-Hall insulating phase in α-RuI_3_, possibly providing insight into experimental reports of bad metallic behavior and sample-to-sample variation in the experimental transport properties of α-RuI_3_.
- Han et al. [12] investigated carrier-doped Van der Waals layered CrSX (X = Cl, Br, I) ferromagnets using first-principles calculations and Monte Carlo studies of possible spin states. Hole doping is predicted to raise the Curie temperature (T_C_) up to 305 K, 317 K, and 345 K for CrSCl, CrSBr, and CrSI, respectively, and increases the magnetic anisotropy energy (MAE) to maximum values of 57, 133, and 1597 μeV/u.c. with spin orientation along the (001) direction.
- Li et al. [31] modeled transition group atomic chains (V, Cr, Mn, Fe, Co, Ni) introduced into nonmagnetic monolayer MoS_2_ with a 4|8ud-type grain boundary. Based on their first-principles calculations using VASP, V chains were predicted to exhibit good thermodynamic stability and self-assemble along the grain boundary. The formation of V, Cr, Mn, and Ni atomic chains successfully induces magnetism into the MoS_2_ system through localized *d*-*d* and *p*-*d* orbital interactions.
- Castaño et al. [25] modeled the excitonic states in Al_x_Ga_1−x_As quantum wells incorporating models for impurities, external electric/magnetic fields, and varying well widths. Solving the one particle Schrödinger equations for the electrons and holes in effective mass approximation via a novel finite element method (FEM) and showing agreement with numerical integration method with a COMSOL v5.6-based calculation predicts that quantum confinement enhances exciton binding in narrow wells, while donor impurities and electric fields reduce it via spatial separation.
- Galanakis [9] reviewed Heusler materials and the formulation of the Slater–Pauling rules for determining their magnetic behavior. The rules have been confirmed by referenced ab initio electronic structure calculations, but the arguments here are qualitative. The review cites the Slater–Pauling rules for all classes of Heusler compounds and how to use them to predict the total spin magnetic moment from counting of levels in the constituent atoms, based mainly on decades of work by the author and coworkers.
- Using a particle swarm optimization (PSO) algorithm with the capabilities of the CALYPSO suite of codes, Xie et al. [36] predicted the global minimum configuration of the trihedral metallo-borospherene *C_3v_* A_1_-Sc_3_B_16_^+^, in which the B_16_-framework comprises unequal B_6_ and B_7_ triangles, forming three equivalent η^7^-B_7_ rings centered with Sc atoms. A second low-lying A_2_ isomer features three equivalent octacoordinated Sc atoms within η^8^-B_8_ rings, both showing high kinetic and thermodynamic stability.
- Sánchez & Wang [35] studied alternating current (AC) transport in nanowires with nearly flat electronic bands using a tight binding model taking account of the 3D structure and a unitary transformation, which expresses it as a set of one-dimensional tight binding models along the wire. The model obtained is thus used to evaluate AC conductivity with the Kubo–Greenwood formula. The results predict a large AC conductivity sensitivity to boundary conditions.
- Porsev & Evarestov [17] comprehensively reviewed the theoretical description, using DFT, MD and TB methods, of one-dimensional nanohelicenes (graphene spirals). The review shows that unique optical, electronic, and magnetic properties are expected to be highly dependent on axial/torsion strains and peripheral modifications of the ribbon. The experiments are also reviewed. A classification nomenclature based on a group theoretic analysis is proposed to categorize all nanohelicenes as modifications of prototype classes.
- Oliver & Huang [14] reviewed computational and experimental research using metal–organic frameworks (MOFs), focusing on the catalytic reaction mechanisms of Zr-MOFs for the hydrolytic degradation of nerve agents (GB, GD, VX) and the photooxidative degradation of sulfur mustard (HD) based on first-principles structure–property relationships.
- Zhou et al. [21] proposed utilizing the Burstein–Moss (BM) effect to achieve multicolor tunability in electrochromic (EC) materials. The BM effect is a blue shift of the optical absorption spectrum of degenerately doped semiconductors due to doping of electrons in the valence band. Selecting bulk Y_2_CF_2_ as a model demonstrates that the BM effect enables EC devices to selectively modulate near-infrared and visible-light absorption with a simplified structure. Calculations use VASP and standard expressions for interband dielectric response.

**Theme 2: Energy Storage, Thermoelectrics, and Photovoltaics**

The following publications applied atomistic modeling to overcome efficiency, safety, and transport bottlenecks in clean energy devices.

- Employing first-principles dynamics, Kim et al. [13] probed amorphous Germanium (Ge) anodes for multivalent-ion batteries. They used a liquid-quench method they had previously reported for constructing amorphous structures from DFT data. They found that although alloying Al and Zn in Ge is thermodynamically unstable, Mg and Ca alloys form stable compounds (Mg_2.3_Ge and Ca_2.4_Ge) with high capacities of 169 mAh/g and 1771 mAh/g, respectively. The Mg-Ge system exhibits a 150% smaller volume expansion ratio (231% vs. 389%) and three orders of magnitude higher ion diffusivity (3.0 × 10^−8^ vs. 1.1 × 10^−11^ cm^2^/s) compared to Ca-Ge, comparable to monovalent ions (Li^+^, Na^+^, K^+^).
- Nguyen et al. [5] used molecular dynamics (MD) to study the effect of three zwitterionic (ZW) molecules (ChoPO_4_, ImSO_3_, and ImCO_2_) on the dissociation of Na^+^ and K^+^ coordination with ethylene oxide (EO) chains in polymer electrolytes. The model contained classical coulomb and 6–12 potentials parametrized by use of the Ligpargen web server. Results predict that ChoPO_4_ will possess the highest cation–EO dissociation ability, while ImSO_3_ will exhibit the lowest, correlating with cation–ZW coordination strength. ImCO_2_ is predicted to enhance Na^+^ diffusion by 20%, while ChoPO_4_ and ImSO_3_ will reduce it by 10%.
- Employing DFT simulations, Marfoua & Hong [4] demonstrated that the bulk semimetallic Janus structure in ZrSeTe exhibits anisotropic transport properties, acting as a good thermoelectric material when the current flows in the out-of-plane direction and as a good coolant when it flows in the in-plane direction. Van der Waals interactions were taken into account using the Grimme DFT-D3 method, and thermal conductivity was evaluated using Phonopy software. The calculations predicted a maximum figure of merit (ZT) of ~0.065 in the out-of-plane direction at 300°, which is one order of magnitude larger than that in the in-plane direction (ZT ~0.006). The in-plane direction shows a cooling performance of 13 W/m·K and a coefficient of performance (COP_max_) of ~90 with a temperature difference (ΔT) of 30 K, allowing out-of-plane current from power generation to be utilized as an in-plane source for active heat pumping.
- By employing the Finite-Difference Time-Domain (FDTD) approach, Cao et al. [24] describe the design of a complete fabrication process for production of ZnO/Au heterojunction nanostructures on silicon-based solar cells, and results of its experimental implementation are reported. To evaluate and optimize the light-absorption properties of the resulting structures, they employed previously developed model and implemented in FDTD. Optimized cell structures improve light absorption efficiency by up to 13.2%, 35.01%, and 63.78% compared to double grating, surface silicon nanostructure, and traditional planar solar cells, respectively, reaching a short-circuit current density of 40 mA/cm^2^ and a power conversion efficiency of 20.17%.
- Using first-principles calculations, Gutsev et al. [10] explored the oxidative properties and charged Frenkel defect formations of halide-mixed perovskites (α-CsPbI_2_Br and β-CsPbI_2_Br). The motive is to improve the stability of CsPbI_3_ by adding Br, but that has proved difficult experimentally because small amounts of Br reduce stability instead of increasing it. The calculations found that nanoinclusions of bromine-rich phases in the α-phase induce lattice strain, accelerating oxidation. Rotation of PbI_4_Br_2_ octahedra are predicted to allow a transition to the equatorial β-phase, which is resilient against hole oxidation (oxidation energy > 0) and demixing (mixing energy < 0), enhancing photostability. However, the authors point out that producing pure β-CsPbI_2_Br experimentally may prove difficult.
- Pavoni et al. [16] investigated twelve systems of monoclinic, orthorhombic, and cubic polymorphs of HfO_2_ doped with yttrium (Y) at 0%, 8%, 12%, and 16% concentrations using Quantum Espresso. When the Y content does not exceed 12%, a stabilization of the cubic phase fraction and an increase in the dielectric constant are observed, with optical bandgaps showing a slight increase as the doping percentage grows. Calculated properties generally are reported to agree well with known experimental values.

**Theme 3: Gas Sensing and Environmental Monitoring**

The following papers focused on investigating the unique surface chemistry and electronic properties of two-dimensional (2D) materials for environmental monitoring and hazardous gas detection.

- Motivated by the possibility that monolayers of SnP_3_ might be used to detect H_2_S, Cui et al. [8] investigated transition metal (Cu, Ag, Zn, Cd)-modified SnP_3_ monolayers using DFT. While Cu-modified SnP_3_ was predicted to exhibit chemisorption with an adsorption energy of −0.749 eV, Zn-modified SnP_3_ was expected to show strong physisorption (adsorption energy of −0.639 eV with a band gap change rate of −28.52% and a rapid recovery/desorption time of 0.064 s at 298 K).
- Nations et al. [33] used DFT and nonadiabatic molecular dynamics (NAMD) to study defect-dependent electronic structures and gas-sensing in cubic α-CsPbI_3_. Among intrinsic defects, the I_Pb_ and Pb_I_ antisite defects are predicted to have transition energy levels near the middle of the band gap, which function as deep traps. Short-term adsorption of NH_3_ selectively coordinates with Pb at Pb_I_ sites and Cs at I_Pb_ sites, significantly altering recombination pathways and accelerating recombination under ambient conditions while suppressing it at higher temperatures and suggesting that the material may be a useful NH_3_ sensor in some industrial applications.
- Qiang et al. [34] designed Pd-decorated V_2_O_5_ porous silicon composites synthesized via magnetron sputtering. First-principles calculations combined with experimental measurements show that the decoration of Pd nanoparticles significantly improves the sensing capabilities, yielding a 3.1-fold increase in response to specific NO_2_ concentrations (ppm level) compared to undecorated sensors, along with rapid response/recovery characteristics, reproducibility, and long-term stability at room temperature.
- Using global structure search and DFT, Jiang et al. [28] predicted a stable planar quasi-isotropic beryllium trinitride (tetr-2D-BeN_3_) monolayer containing pentagonal, hexagonal, and dodecagonal rings alongside “S”-shaped N_6_ polymeric units. The material exhibits a high energy density of 3.34 kJ/g, an indirect band gap of 2.66 eV, a low in-plane Young’s stiffness (52.3–52.9 N/m), a high Poisson’s ratio (0.55–0.56), and exceptional selectivity for helium/argon gas separation, achieving a maximum selectivity up to 10^23^ (He/Ar).

**Theme 4: Advanced Sensing and Quantum Metrology**

The following research areas highlighted the development of highly sensitive, nanoscale platforms for mechanical, chemical, and optical detection.

- Based on DFT calculations, Paudel et al. [3] explored the pressure dependence of optical spectra of nitrogen-vacancy (NV) defects in diamond. The DFT results are used to parametrize a low-energy Hamiltonian spin model to determine the effect of strain on the defect band edges and band gaps by varying the lattice parameters of a diamond supercell hosting a single NV center. The developed Hamiltonian includes the effect of stress on the energy level of a ±1 spin manifold at the ground state, predicting quantum pressure sensing of up to 0.3 MPa/$\sqrt{Hz}$ using the experimentally measured spin dephasing time. The detectable energy shifts per unit pressure by these methods were found to surpass those of traditional optical sensors by several orders of magnitude, suggesting the potential usefulness of such NV centers in diamond for pressure determinations under harsh industrial and natural environments.
- Lv et al. [32] studied a proposed metal-dielectric device as a potential refractive index sensor. Called a “yurt tetramer” it consists of four cylinders with rounded caps. The cylinders and caps are supposed to consist of adielectric Si_3_N_4_ sitting on a planar slab of gold (Au). The entire structure is a few 100 nm on each side. It was analyzed by taking dielectric response functions of the bulk materials from the literature for the components and simulating the electromagnetic response of the system to an EM plane-wave incident, normally incident from below using the finite-difference time-domain (FDTD) method, as implemented in the Ansys Lumerical FDTD 2023 R2.1 software system. The device is predicted to achieve high polarization insensitivity, multi-band tunability across visible to near-infrared spectra, and a narrow linewidth. A surface plasmon resonance and two Fano resonance modes at 37.43 nm, 808.99 nm, and 939.50 nm are predicted to yield an enhanced light–matter interaction with a maximum refractive index sensitivity of 500.94 nm per refractive index unit and a Q-factor of 500.94.
- Cheng et al. [26] analyzed the optical response of three kinds of rotationally symmetric Au-Ag nanoparticles in randomly orientations using a T-matrix approach due to Mishchenko et al. Nanorods were determined to have the largest response to the embedding dielectric medium. Optimizing with respect to gold (Au) molar fraction, aspect ratio and size for high-performance optical biosensing, they predict a refractive index sensitivity of 395.2 nm per refractive index unit and an optimized figure of merit of 7.16 for the best nanorods.
- Employing the Lumerical FDTD approach, Li et al. [30] characterized the far-field and near-field optical magnetic responses of a gold-oblate nano-ellipsoid on gold mirror (ONEOM) structure. They found a pronounced Purcell factor enhancement for magnetic dipole radiation by introducing magnetic dipoles into the gap between the nano-ellipsoid and the mirror in the ONEOM structure. By optimizing with respect to gap size and structural parameters, they predicted a 230-fold maximum magnetic radiation Purcell factor enhancement for the structure, suggesting that it may be useful for single-photon sources, nonlinear frequency conversion, and plasmonic sensing.

**Theme 5: Mechanical Stability, Strain, and Material Properties**

The following publications referred to the fundamental physics associated with structural durability, phase transitions, and strain-induced effects in nanomaterials.

- Employing MD (LAMMPS-2018) with a reactive force field potential (Reaxff), Zhang et al. [38] investigated fracture toughness of boron carbide (B_4_C), proposed for use in ceramic armor. The strength disparity on either side of a twin boundary is shown to impede crack propagation. At an angle of 120° between the twin boundary and the prefabricated crack, the boundary provides a maximum toughening effect, enhancing the toughness by 32.72%, whereas standard phase boundaries have no toughening effect.
- Using MD, Bai et al. [23] simulated atomic displacement cascades in InP induced by 1–10 keV primary knock-on atoms (PKAs). They improved existing analytical bond-order potentials (ABOPs) for the force fields by adding an account of electronic stopping (ES) and tested the new potentials using DFT calculations. They found that ES is predicted to reduce stable point defects by 32.2% and 27.4% for 10 keV In-PKA and P-PKA, respectively, weakening the damage regions and causing medium-to-large defect clusters to vanish in favor of small clusters. Incorporating electronic stopping (energy loss) reduces stable point defects by 32.2% and 27.4% for 10 keV In-PKA and P-PKA, respectively, weakening the damage regions and causing medium-to-large defect clusters to vanish in favor of small clusters.
- Yan et al. [37] investigated the band structure and electronic effective mass of InP under different strains using DFT implemented in the Quantum ATK software package. Both uniaxial and biaxial tensile strain are predicted to have linear effects on band gap values, while the band gap transitions to a metallic state under hydrostatic pressures (negative tensile strain) below −7 GPa. Strain engineering is also predicted to modify effective mass and induce anisotropy in electron mobility.
- Using DFT and a fit to the band structure using energy-dependent effective masses (Rudenko model), Chung & Chang [27] explored the carrier transport in monolayer HfS_2_ under biaxial strain. Mobilities were calculated from the fitted model using the Kubo–Greenwood formula. Biaxial compressive strain is predicted to reduce the effective mass of the conduction band and the phonon scattering rate, leading to a ~90% increase in electron mobility and a ~13% increase in hole mobility at a 4% compressive strain.
- Employing DFT, Tian et al. [18] simulated epitaxially grown (001) BiCoO_3_ films. Tensile strains above 3.922 Å are predicted to induce a magnetic phase transition in the ground state from C-type to G-type antiferromagnetic ordering. Larger tensile strains should induce a continuous structural phase transition combining ferroelectric and antiferrodistortive modes, allowing a “Type Zero” isostructural phase transition.
- Using the modified couple stress theory and scale distribution theory (MCST&SDT), Jiang et al. [29] worked out in detail to analyze vibrations in functionally graded porous Cu-Si microcantilevers in fluids with applications to micro-electro-mechanical systems (MEMS) in mind. Four different pore distributions are considered. System sizes are of the order of microns and materials properties are taken from the literature. The presence of pores reduces Young’s modulus and the quality factor. The effect of porosity on the resonant frequency is negatively correlated when the gradient factor (n) is small (n < 1) but positively correlated when the gradient factor is large (n > 1).
- Using plane-wave DFT calculations, Vig et al. [19] investigated size-dependent cohesive energies of 3*d*, 4*d*, and 5*d* transition-metal nanoclusters. They found that cohesive energies decrease with decreasing size due to increased surface-to-volume ratios and quantum confinement. Nanoclusters with fewer layers exhibit lower cohesive energies, with monolayer nanoclusters showing the lowest stability.
- Employing DFT, Zhang et al. [20] evaluated high-index facets of bismuth vanadate (BiVO_4_) for water splitting. The results indicate that the high-index facets—such as (012), (210), (115), (511), (121), (132), and (231)—will show an n-type behavior and possess band edges capable of producing O_2_ without an external bias, displaying lower overpotentials than conventional (010) and (110) surfaces.

**Theme 6: Advanced Computational and ML Methodologies**

The following research highlighted emerging machine learning (ML), neural network, and quantum computing techniques for complex material analysis.

- Achar et al. [7] developed “DeepCDP”, a deep neural network ML formalism that predicts charge densities in molecules and condensed phase systems using only atomic positions. Trained on DFT results for bulk copper, LiF, silicon, water, and 2D charged/uncharged graphene, the model scales linearly with system size, are highly parallelizable, and accurately tracks charge (proton) locations at a significantly reduced computational cost.
- Using Green–Kubo MD with a deep machine learning potential (GK-DP) combined with BTE-DFT, Han et al. [11] investigated the thermal conductivity of monolayer InSe. While standard BTE-DFT overestimates thermal conductivity at 13.08 W/mK by excluding four-phonon scattering, the GK-DP approach predicts 9.52 W/mK at 300 K, in excellent agreement with experimental values.
- Employing a DFT SCAN (meta-GGA) functional paired with PseudoDojo pseudopotentials, Pavoni et al. [15] evaluated the electronic and optical properties of monoclinic MoO_2_ and orthorhombic MoO_3_. Calculated band gaps, densities of states, and optical spectra show excellent agreement with the experimental literature, demonstrating that SCAN accurately reproduces the bonding characteristics of molybdenum oxides.
- Alfonso et al. [22] utilized a quantum-classical active space-embedding approach on a quantum computer simulator (requiring up to 14 qubits) to map reaction profiles and barriers for the hydrogenation of small lithium clusters (Li_2_, Li_3_, Li_4_). The predicted potential energy curves and reaction barriers compare well with advanced classical quantum chemistry methods, establishing a computational benchmark for quantum chemistry calculations.

**Future Research Directions**

Based on the 36 publications collected in these Special Issues, several highly promising avenues for future theoretical and experimental research have emerged. Here, we point out five main possible directions.

1. **Scaling and Hybrid Modeling (ML + DFT, quantum computing):**
  ○ *Direct Gap Bridging:* While deep neural networks like “DeepCDP” can accurately predict charge densities, future work should focus on integrating these models directly into AIMD loops. This would allow for the simulation of larger, mesoscopic systems (closer to the 10 nm limit) without sacrificing the accuracy of first-principles calculations. This is a very promising direction and can be expanded to allow computation of other properties of many electrons systems.
  ○ *Universal ML Potentials:* Expanding deep potentials (such as the GK-DP model used for thermal transport) [7] to multi-component, highly disordered, or amorphous interfaces (like polymer electrolytes with additives or amorphous Ge anodes) could significantly accelerate the discovery of battery and thermoelectric materials.
  ○ *Quantum computing:* With the rapid development of quantum computers, quantum computing may leverage the principles of quantum mechanics to process complex problems exponentially faster than classical computers. However, the limited number of qubits currently available limit quantum chemistry calculations on quantum computers or simulators to small systems [22]. With the arrival of a new generation of quantum hardware, quantum computing for computational chemistry and materials will allow researchers to calculate the ground-state energy and dynamics of molecules with high precision and linear or polynomial scaling, because the quantum computers naturally represent the wave functions of electrons.
2. **Quantum Engineering of Defect-State Sensors:**
  ○ *Multi-functional Quantum Sensing:* The use of NV centers in diamond for pressure sensing can be expanded to multi-parameter sensing (simultaneous detection of temperature, magnetic field, and strain) under extreme subsurface or high-temperature superconducting environments. This will require the development of more complex, strain-coupled low-energy Hamiltonians.
3. **Real-world Environment Simulations for Gas Sensors:**
  ○ *Humidity and Competitor Gas Interferences:* Computational modeling of gas sensors (like SnP_3_, [8] CsPbI_3_, [33] and V_2_O_5_ [34]) must transition from ideal vacuum/gas setups to complex, multi-component gas environments. Modeling the co-adsorption of water vapor, oxygen, and carbon dioxide at first-principles levels will help design sensors with higher selectivity and fewer false-positive readings.
4. **Optimizing Solid Polymer Electrolytes (SPEs) and Multi-Valent Anodes:**
  ○ *Design of Dynamic Zwitterionic Additives:* Future simulations should screen a broader library of zwitterionic structures to find molecules that maximize cation–EO dissociation while minimizing the coordination-induced deceleration of cation diffusion.
  ○ *Interface Engineering of Amorphous Anodes:* Exploring the interfaces between amorphous Ge anodes and solid-state electrolytes at the atomic scale will be crucial for predicting dendrite growth and mechanical degradation during cycling in multivalent-ion batteries.
5. **Validation and Understandability**:
  ○ As many-body simulation becomes more and more dependent on legacy and commercial codes, the validity of published results can become more difficult to determine. Many workers are understandably not familiar with all the details of the software packages with which they work, and the precision and validity of results are therefore difficult to determine for the workers themselves, the readers of their papers, engineers who depend on the device design results, and experimentalists designing the experiments. One way to approach this issue is to estimate uncertainties by carrying out studies using more than one, preferably several, software platforms at the same length and time scale, but this is seldom done before publication. There are other possible approaches to estimating uncertainties in simulation results, and the development, refinement and use of such approaches, as well as the quantitative reporting of the uncertainties (e.g., error bars) along with the results, are encouraged.

## Figures and Tables

**Table 1:** Summary of articles published in these Special Issues [1,2].

Theme	Topic	Method and Software	Ref.
Spintronics and Correlated Electronic Structures	Kitaev Magnet Candidates α-RuCl_3_ and α-RuI_3_	DMFT, eDMFT, WIEN2K	[6]
	Hole-Doped Ferromagnets CrSX (X = Cl, Br, I)	DFT-D3, AIMD, Phonopy, VAMPIRE, VASP	[12]
	MoS_2_ Transition Metal Chains	DFT, GGA-PPE, VASP	[31]
	Quantum Well Excitons	FEM, COMSOL	[25]
	Slater-Pauling Rules in Heusler materials	Review	[9]
	Spherical Trihedral Metallo-Borospherenes	PSO, DFT, Quantum Espresso	[36]
	Flat-Band AC Transport in Nanowires	Kubo–Greenwood formula	[35]
	Computational Modeling of Nanohelicenes	Review	[17]
	Removal of Chemical Warfare Agents	Review	[14]
	Burstein–Moss Electrochromics	DFT, GGA-PBE, HSE06, VASP	[21]
Energy Storage, Thermoelectrics, and Photovoltaics	Multivalent-Ion Batteries	DFT, VASP	[13]
	Zwitterionic Battery Additives	MD, Gromacs	[5]
	Bifunctional ZrSeTe Janus Thermoelectrics	DFT-D3, VASP	[4]
	ZnO/Au Silicon Solar Cells	FDTD	[24]
	APb(I_1−x_Br_x_)_3_ Photostability	DFT-D3, VASP	[10]
	Y-Doped HfO_2_	DFT, Q-ATK, Quantum ESPRESSO	[16]
Gas Sensing and Environmental Monitoring	SnP_3_ Monolayers	DFT-D, DMol	[8]
	NH_3_ Sensing in Perovskites	DFT, mGGA-SCAN, VASP	[33]
	NO_2_ Gas Sensors	DFT-D3, VASP	[34]
	BeN_3_ Energetic Materials	DFT, HSE06, VASP, CALYPSO	[28]
Advanced Sensing and Quantum Metrology	Quantum Pressure Sensors	DFT, VASP	[3]
	Yurt Tetramer Metasurfaces	FDTD	[32]
	Au-Ag Alloy Biosensors	T-matrix	[26]
	Magnetic Purcell Enhancement	FDTD	[30]
Mechanical Stability, Strain, and Material Properties	Nanotwinned Boron Carbide	MD, LAMMPS	[38]
	Radiation Damage in Indium Phosphid (InP)e	MD, DFT, LAMMPS, VASP	[23]
	Strain Engineering in InP	DFT, Quantum ATK	[37]
	Carrier Mobility in HfS_2_	DFT, Quantum ATK	[27]
	BiCoO_3_ Phase Transitions	DFT, VASP	[18]
	Porous Microcantilever Vibrations	MCST, SDT	[29]
	Size Effects on Cohesive Energies	DFT, VASP	[19]
	High-Index BiVO_4_ Facets	DFT, VASP	[20]
Advanced Computational and ML Methodologies	Deep Charge Density Prediction	DFT-MD	[7]
	ML Potential for InSe	DFT, MD, DeePMD-kit, VASP	[11]
	MoO_2_ and MoO_3_ SCAN Calculations	DFT, Quantum ATK	[15]
	Quantum Active Space-Embedding	DFT, VQE, PySCF	[22]
